# Characterization of Expression Quantitative Trait Loci in Pedigrees from Colombia and Costa Rica Ascertained for Bipolar Disorder

**DOI:** 10.1371/journal.pgen.1006046

**Published:** 2016-05-13

**Authors:** Christine B. Peterson, Susan K. Service, Anna J. Jasinska, Fuying Gao, Ivette Zelaya, Terri M. Teshiba, Carrie E. Bearden, Rita M. Cantor, Victor I. Reus, Gabriel Macaya, Carlos López-Jaramillo, Marina Bogomolov, Yoav Benjamini, Eleazar Eskin, Giovanni Coppola, Nelson B. Freimer, Chiara Sabatti

**Affiliations:** 1 Department of Health Research and Policy, Stanford University, Stanford, California, United States of America; 2 Department of Psychiatry and Biobehavioral Sciences, University of California Los Angeles, Los Angeles, California, United States of America; 3 Center for Neurobehavioral Genetics, University of California Los Angeles, Los Angeles, California, United States of America; 4 Department of Human Genetics, University of California Los Angeles, Los Angeles, California, United States of America; 5 Department of Psychiatry, University of California San Francisco, San Francisco, California, United States of America; 6 Cell and Molecular Biology Research Center, Universidad de Costa Rica, San Pedro de Montes de Oca, San José, Costa Rica; 7 Grupo de Investigación en Psiquiatría (Research Group in Psychiatry (GIPSI)), Departamento de Psiquiatría, Facultad de Medicina, Universidad de Antioquia, Medellín, Colombia; Mood Disorders Program, Hospital San Vicente Fundacion, Medellín, Colombia; 8 Faculty of Industrial Engineering and Management, Technion, Haifa, Israel; 9 Department of Statistics and Operations Research, Tel Aviv University, Tel Aviv, Israel; 10 Department of Computer Science, University of California Los Angeles, Los Angeles, California, United States of America; 11 Department of Biomedical Data Science and Department of Statistics, Stanford University, Stanford, California, United States of America; Perelman School of Medicine, University of Pennsylvania, UNITED STATES

## Abstract

The observation that variants regulating gene expression (expression quantitative trait loci, eQTL) are at a high frequency among SNPs associated with complex traits has made the genome-wide characterization of gene expression an important tool in genetic mapping studies of such traits. As part of a study to identify genetic loci contributing to bipolar disorder and other quantitative traits in members of 26 pedigrees from Costa Rica and Colombia, we measured gene expression in lymphoblastoid cell lines derived from 786 pedigree members. The study design enabled us to comprehensively reconstruct the genetic regulatory network in these families, provide estimates of heritability, identify eQTL, evaluate missing heritability for the eQTL, and quantify the number of different alleles contributing to any given locus. In the eQTL analysis, we utilize a recently proposed hierarchical multiple testing strategy which controls error rates regarding the discovery of functional variants. Our results elucidate the heritability and regulation of gene expression in this unique Latin American study population and identify a set of regulatory SNPs which may be relevant in future investigations of complex disease in this population. Since our subjects belong to extended families, we are able to compare traditional kinship-based estimates with those from more recent methods that depend only on genotype information.

## Author Summary

We assess the heritability and genetic regulation of gene expression in a population of 786 individuals from Costa Rica and Colombia. The subjects, originally recruited in a study of bipolar disorder, are related within 26 extended families. This design allows us to compare estimates of the heritability of gene expression obtained using both traditional and genotype-based methods. We address questions regarding the architecture of genetic regulation including the extent to which gene expression is influenced by variants located nearby vs. far away on the genome and how many variants affect the expression of a given gene. In addition, we identify genetic variants which regulate gene expression; these serve as candidates for future studies to establish the genetic basis of complex traits, including those related to bipolar disorder, and also provide insight into the architecture of genetic regulation in this unique Latin American study population.

## Introduction

Dozens of investigations have now shown that the identification of local eQTL may play a crucial role in delineating the causal variant(s) contributing to genetic associations observed for complex disorders or quantitative traits [1–3]. While it may be particularly informative to evaluate, for a given trait, eQTL specific to tissues implicated in the manifestation of that trait, this strategy may be infeasible on a large scale for human brain related traits, such as psychiatric disorders and their endophenotypes. In this study we report the results of gene expression in lymphoblastoid cell lines (LCL) for 786 genotyped members of Costa Rican and Colombian pedigrees [4–5]. While the subjects in this study were originally recruited as part of an investigation for severe bipolar disorder (BP1), we found no relationship between the observed gene expression data and BP1 (S1 Text). We selected LCL for ease of study and on the basis of the increasing evidence that a substantial proportion of local genetic regulation is conserved across tissues [6–8]. While distal regulation has been found to have a higher degree of tissue-specificity vs. local regulation [9], it is unclear to what extent this finding reflects the very limited power to detect distal associations in a given tissue and resulting underestimates for the extent of overlap across tissues. Studying LCLs has enabled at least a partial reconstruction of the specific regulatory network (i.e. the bipartite graph relating genetic variants to gene expression traits, the strength associated to each of these edges, and the overall impact of genetic variants on the variability of expression) for these families, allowing us to identify those components that might show differences from the general population. We study the genetic regulation of expression in these pedigrees at a multiscale level: we estimate heritability, evaluate the relative importance of local vs. distal genomic variation, identify variants with regulatory effects, and analyze the role of multiple associated SNPs in the same region. By capitalizing on known pedigree structure, as well as extensive genotyping, we can compare different methodologies for heritability estimation. The most interesting element of regulatory networks for our purpose is the localization of SNPs with regulatory effects (eSNPs): these variants are candidates for future studies investigating association to the BP1 endophenotypes measured in our sample, and also provide insight into functional genetic variation in this unique population. To control the rate of false discoveries of eSNPs, we adopt a novel hierarchical testing procedure that leads to the analysis of expression quantitative trait loci (eQTL) data in a stage-wise manner with increasing levels of detail.

## Methods

### Sample collection

The study subjects are members of 26 Costa Rican and Colombian pedigrees ascertained from local hospitals and clinics based on multiple individuals affected with BP1. Descriptions of pedigrees and ascertainment procedures are provided in [4]. Written informed consent was obtained from each participant, and institutional review boards at participating sites approved all study procedures (UCLA Medical Institutional Review Board 3 [IRB # 11–000407]; Scientific Ethics Committee of the University of Costa Rica [Project No. 801-91-552]; and the Bioethics Committee of the Institute of Medical Research, University of Antioquia [Project Name “Genética de la enfermedad Bipolar. Endofenotipos bipolares en una población aislada genéticamente”]).

### RNA extraction and measurement of gene expression

Lymphoblastoid cell lines (LCLs) were established at two sites. RNA was extracted from these cell lines and its expression quantified using Illumina Human HT-12 v4.0 Expression BeadChips. Expression values were background corrected, quantile normalized, log2 transformed, and corrected for major known batch effects. The outcome of these procedures is what we refer to as ‘probe expression’ for all subsequent analyses. After quality control filters, the 34,030 probes included in the final set were aligned to at most 2 locations in hg19, contained no common SNPs (as defined in dbSNP 137 or 138), their expression was detected in at least one individual, and queried 24,385 unique genes. As discussed in S1 Text, both the choice of normalization procedure (across all subjects rather than within pedigree) and of detection threshold (which is fairly generous) affect downstream estimates including expression heritability. For a detailed description of the processing steps used at each site and the RNA quantification, normalization, and quality control procedures, see S1 Text.

### DNA extraction, genotyping, and subject inclusion criteria

DNA was extracted from blood or LCLs using standard protocols. Illumina Omni 2.5 chips were used for genotyping, in three batches. A subset of samples was repeated in each batch to enable concordance checks. A total of 2,026,257 SNPs were polymorphic and passed all QC procedures, including the evaluation of call rate, testing for Hardy Weinberg equilibrium, and Mendelian error. A total of 1,024,051 autosomal SNPs with minor allele frequency (MAF) of at least 10% were selected for use in the subsequent analysis. A threshold of 10% was chosen since we have very limited power to detect SNP-gene associations for SNPs with MAF < 10% given the size of our study population. We would like to note, however, that this threshold may result in lower estimates for the local portion of gene expression heritability and possibly different numbers of independent local eSNPs vs. studies using a less stringent threshold. After excluding married-ins with no descendants in the study and cases of possible contamination, the analyzed sample contains 786 individuals with both genotype and gene expression data. (See S1 Text for details.)

### Adjustment for factors affecting global gene expression

In order to adjust for both known and unknown factors affecting global gene expression, all association and heritability analyses include age, sex and batch as covariates, in addition to a set of PEER factors to adjust for latent determinants of global gene expression [10]. We chose to include 20 PEER factors on the basis of the proportion of global gene expression explained, and found that these PEER factors were strongly correlated with batch, but not with family groupings, suggesting that they are in fact correcting for technical artifacts.

### Local vs. distal genetic regulation

The eQTL literature documents a distinction between cis vs. trans regulation, although the precise definition of these is sometimes elusive. Following the suggestion of [3], we adopt the terminology “local” and “distal” regulation to distinguish the situations where genetic variants and the genes whose expression they regulate are nearby or far away in the genome, without any assumption on the mechanisms of this regulation. Operationally, we define “local” associations as those between SNPs and probes where the SNP is located within 1Mb of either end of the probe, and “distal” as all other probe-SNP associations, including those across different chromosomes.

### Heritability of gene expression

For each probe, we estimated the heritability of gene expression using two approaches: a variance components model relying on known family relationships as implemented in Mendel [11] and a variance decomposition based on observed genotypic similarities among individuals as implemented in GCTA [12]. Both analyses included age, sex, batch and PEER factors as covariates. In our primary GCTA analysis, we utilized a genetic relatedness matrix (GRM) based on the full set of genome-wide SNPs. This allowed us to calculate the ratio of genetic variability over total phenotypic variability for each probe. We then compared the estimates obtained using Mendel and GCTA. To determine which probes were significantly heritable, we relied on the likelihood ratio test implemented in GCTA to obtain p-values for the significance of the genetic variance component.

To consider the effect of shared environment on the heritability of gene expression, we computed a version of the variance components model in Mendel with a variance component included for effects corresponding to pedigree membership. We also examined correlations between spouses, siblings, and parent-child pairs using the function FCOR from the S.A.G.E. software package [13], which allows the estimation of familial correlations and their asymptotic standard errors [14–15]. Because FCOR does not allow the inclusion of covariates, expression was first regressed on all covariates and the residuals were used for correlation analysis.

To obtain an unbiased estimate for the mean heritability of gene expression, Price et al. [16] allow negative values for heritability. Although Mendel requires heritability estimates to be constrained to [1] interval, GCTA allows this assumption to be relaxed; to assess the impact of this constraint, we compute both constrained and unconstrained estimates in GCTA for the ratio of genetic variability to total phenotypic variability.

As a secondary analysis, we used GCTA to refine the variance decomposition of probe expression to obtain estimates of the proportion of probe heritability due to local regulation. Specifically, we utilized the multiple GRM option in GCTA (which allows partitioning of the phenotypic variance into components explained by different SNP subsets; see for example [17]) with two GRMs specified: one based on the set of SNPs within 1Mb of the probe of interest (whenever a sufficient number of SNPs was present), and one based on all SNPs genome-wide (a reasonable stand-in for relatedness based on distal SNPs). This strategy allowed us to partition the heritability into local vs. global components and calculate the ratio of local genetic variability to total variability.

With regards to interpretation of the resulting estimates, we note that the goal of GCTA is to estimate the additive effects of the genotyped SNPs, rather than a true estimate of heritability. Yang et al. [12] therefore recommend excluding related subjects since including these will bias the estimate of the proportion of variance explained by common variants upward due to factors such as shared environment or rare variants passed down within a family. Since we include related subjects, our GCTA results will be inflated relative to those for unrelated subjects, and therefore are more similar to the family-based heritability estimates. The relatedness of our subjects (and the fact families share a number of environmental factors) is also likely to affect the partitioning of heritability into local vs. distal components in GCTA: since the GRM from local SNPs will align less closely to the correlations due to family structure and shared environment than that of the GRM from genome-wide SNPs, the proportion of genetic variance to due local SNPs may be underestimated.

### Computation of SNP-probe association p-values

We computed association p-values for each SNP-probe pair using the pedigree GWAS option in Mendel including additive genetic and environmental variance components [11,18]. The Mendel implementation relies on a score test to greatly increase the speed of computation of association p-values in mixed models. For the most promising SNP-probe pairs, a standard likelihood ratio test (LRT) is conducted, and effect sizes are derived. In our analysis, we included age, sex, batch and PEER factors as covariates. We performed the LRT for the 100 most significant local and 100 most significant distal SNPs for each probe, with the score test used for the remaining SNP-probe pairs.

### Multiplicity adjustment and identification of significant results

Our hierarchical testing approach is based on the selective procedure by Benjamini and Bogomolov [19] whose effectiveness in genetic association studies for multiple phenotypes in demonstrated through the simulations provided in [20]. A version of this approach tailored to the eQTL context is implemented in the TreeQTL R package [21]; the current work is the first application of the proposed methods to a real-world eQTL study. The testing procedure is designed to take into account that local regulation is more common than distal (the hypotheses in these two classes are tested separately) and that SNPs with distal effects are likely to affect the expression of more than one probe. While the possibility of identifying variants involved in the local regulation of each probe depends on the sample size and the signal strength, it is quite reasonable to expect that the expression of every gene could be affected by appropriate sequence variation in the genomic region surrounding it. In contrast, one expects that only a small portion of the genotyped variants have any regulatory role. Both to capitalize on this heterogeneity and because our ultimate interest is to identify genetic variants that have phenotypic effects, we apply a multiscale testing strategy to first identify SNPs that have regulatory effects (eSNPs). We control the FDR in these discoveries at a target level of 0.05 with the Benjamini-Yekutieli [22] procedure, a conservative approach which is robust to dependence among the test statistics and therefore appropriate given linkage disequilibrium among the SNPs. In a second stage we investigate which specific probes are influenced by these eSNPs. We control the expected average proportion of false SNP-probe associations across the selected SNPs at a target 0.05 level with the Benjamini Bogomolov (BB) method [19], which has been shown to control the relevant error rates under the typical dependency structure of multi-trait GWAS [20]. We adopt this hierarchical multiple testing strategy to improve the interpretability and relevance of our findings, as it controls error rates regarding the discovery of functional SNPs and the association of these SNPs to traits which are not controlled using standard non-hierarchical multiple testing corrections. While our primary goal in adopting the hierarchical testing procedure is to control these important error rates, we are able to take advantage of the heterogeneity across genetic variants (mentioned above) to preserve power to the extent possible.

### Genomic characteristics of eSNPs

We studied the position of local eSNPs relative to the transcription start site (TSS) of the gene queried by the probe to which they were associated. TSS information was derived from the UCSC Genome Browser (http://genome.ucsc.edu/). We investigated the distal eSNPs by assessing their overlap with local eSNPs and by comparing their locations with the annotations derived by the Roadmap Epigenomics Project (http://egg2.wustl.edu/roadmap/web_portal/) for LCLs using ChIP-Seq and DNAse-Seq [23].

### Cross-study comparisons

Cross-study comparisons are hampered by many factors including changes in annotation resulting in different gene symbols, changes in SNP names, and the use of different versions of the human physical map. We downloaded results from eQTL analysis of blood or LCL from the seeQTL database (http://www.bios.unc.edu/research/genomic_software/seeQTL/) [24], including results from [25–26] and a meta-analysis of HapMap LCLs, and also obtained results of [27] for associations with FDR less than 50%. We used official gene symbols to compare results across studies.

### eSNP effect sizes, and percentage of heritability explained

For each probe associated to some of the discovered eSNPs, we constructed a multivariate linear mixed model relating expression to the genotypes at significant SNPs, local or distal. Using the variance components model implemented in Mendel, a fixed effect was estimated for age, sex, batch, the PEER factors, and each of the genetic variants, while a random effect was used to capture family structure. We then calculated the proportion of variance explained in this model by the collection of local eSNPs and distal eSNPs and compared it with the local and global heritability estimates obtained using the partitioning approach of GCTA.

To account for the fact that linkage disequilibrium may lead to the identification of a number of neighboring SNPs as associated to the same probe—even when the underlying association is effectively captured by one SNP alone—we performed model selection to determine the number of SNPs that might reasonably correspond to independent signals. Specifically, after transforming the data to obtain independent observations (using the appropriate variance covariance matrix determined from the mixed model analysis in Mendel), for each probe we carried out stepwise forward selection, relying on the BIC criteria, and using residual expression (adjusted for covariates) as the response and the eSNPs associated to the probe as the pool of predictors. This procedure gave us an estimate of the number of independent eSNPs affecting each probe, as well as the value of the percentage of variance explained (the adjusted r^2^ value) for the resulting multivariate linear model. For comparison, we also obtained the percentage of variance explained (the r^2^ value) for the univariate linear model using the most strongly associated eSNP (local or distal) as the only predictor. We then computed the ratio of the r^2^ for each model to the heritability previously estimated using the variance components model in Mendel.

## Results

### Heritability of gene expression

**Fig 1:**
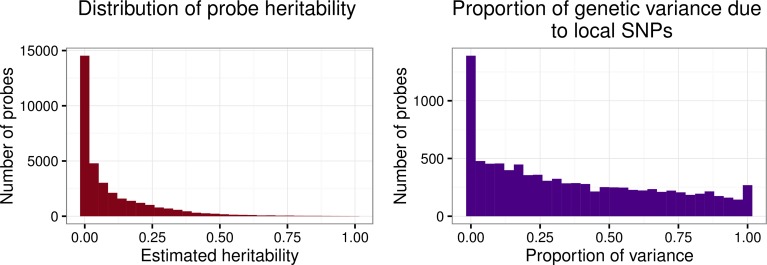
Expression heritability and proportion of genetic variance due to local effects. Distribution of estimated heritability of probe expression obtained using Mendel for all 34,030 probes (left), and distribution of the proportion of total genetic variance attributed to local genetic variation (right) for the 9,458 significantly heritable probes (FDR<0.05) where partitioning using the multiple GRM approach in GCTA was possible.

The distribution of heritability estimates across all 34,040 probes obtained using Mendel, shown at left in Fig 1, had median 0.03 (mean = 0.10). Estimates of the heritability of gene expression based on kinship obtained using Mendel correlated well with estimates of the proportion of phenotypic variation due to genome-wide SNPs obtained using GCTA (r = 0.99), suggesting agreement between the known pedigree structure and levels of genetic similarity in the subjects (S1 Fig); the estimates from Mendel tended to be slightly larger than those from GCTA, particularly for values closer to 1. The median proportion of variance explained by genome-wide SNPs as computed by GCTA was 0.04 (mean = 0.10) when constrained to the [1] interval, while the unconstrained estimates had median 0.03 and mean 0.09 (S1 Fig). The likelihood ratio test for the significance of the genetic variance component in GCTA resulted in 12,631 rejections (37%) at p<0.05; 10,630 rejections (31%) at FDR threshold 0.05; and 4,496 rejections (13%) applying the Bonferroni correction to target FWER 0.05. The median proportion of variance in gene expression explained by genome-wide SNPs among probes satisfying FDR<0.05 was 0.22 (range 0.07–1.00) when constrained to the [1] interval, while the unconstrained estimates for these probes was 0.23 (range 0.07–1.01).

The inclusion of a family variance component in Mendel corresponding to pedigree membership reduced the heritability estimates from a median of 0.034 (mean = 0.10) to a median of 0.026 (mean = 0.09). The GCTA estimates of the proportion of phenotypic variability explained by genome-wide SNPs are most similar to the Mendel kinship-based estimates of heritability without the additional family variance component, suggesting that GCTA functions very similarly to kinship-based approaches in a family setting (S1 Fig). In our examination of familial correlations, we found very few spouse correlations (which reflect shared environment but not kinship) to be significant even at the 0.05 level (4%), whereas 23% and 27% of parent-offspring and sibling correlations were significant at this threshold. The median (mean) correlations for the three relationship classes were 0.01 (0.009) for spouses, 0.04 (0.06) for sibling, and 0.03 (0.04) for parent-offspring pairs. Sibling and parent-offspring correlations were well-correlated to the heritability estimates, whereas there was no correlation between heritability and spouse correlations. Together, these two approaches suggest that shared environment accounts for a small proportion of our estimated heritabilities.

Among the 10,630 significantly heritable probes (FDR<0.05), 9,458 had a sufficient number of SNPs in the local region to obtain a GRM usable for partitioning; for these probes, a median of 30% of the total genetic variance was attributed to local genetic variation (mean = 37%). The distribution of the proportion of total genetic variance attributed to local genetic variation for these probes is shown at right in Fig 1. Probes with a low proportion of genetic variance attributed to local genetic variation (<5%) have a significantly smaller number of local SNPs than those with a larger proportion (one-sided t-test p<2.2e-16) and are associated to a significantly higher number of distal eSNPs (one-sided t-test p = 0.02), suggesting that both a failure to measure relevant local SNPs and the effects of distal regulation may explain the fraction of heritable probes found to have a low local proportion of genetic variance.

### eSNP discoveries

QQ plots for the local and distal SNP-gene association analyses are shown in S2 Fig. Taken together, these plots demonstrate that the distribution of the test statistics under the null is as expected, and that there is strong evidence for a significant number of non-null hypotheses genomewide for both local and distal regulation. Controlling the FDR of eSNP discoveries at a 5% level, we identify 139,668 local eSNPs and 11,016 distal eSNPs. Controlling the expected value of the average proportion of false discoveries for probe-SNP association across the discovered eSNPs to 5% as well results in the identification of 305,635 local probe-SNP pair associations and 22,304 distal probe-SNP pair associations. There are 10,065 distinct probes involved in these associations (9,645 in local regulation and 1,081 in distal, with an overlap of 661).

**Fig 2:**
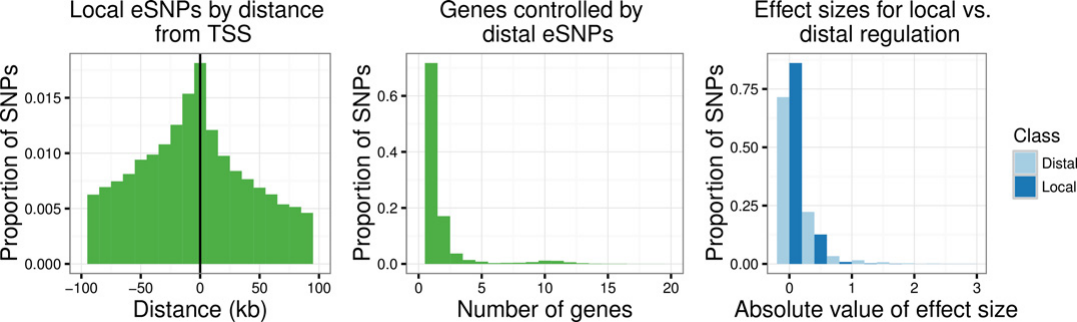
Characteristics of local and distal eSNPs. Position of local eSNPs relative to transcription start site (TSS) of the gene queried by the associated probe (left). Number of genes controlled by distal eSNPs (center), excluding SNP kgp22834062, which was associated to 129 genes. Effect sizes (absolute values of the regression coefficients) of the most significant SNP for each probe with any local or distal associations (right).

We now consider some of the characteristics of the discovered eSNPs. In keeping with current understanding of the mechanisms of local regulation, 72% of the local eSNPs are upstream from the gene they putatively regulate, and 15% of these are within 100kb upstream from the transcription start site (TSS). The distribution of local eSNPs by distance from the TSS, calculated as the TSS position of the queried gene minus the SNP position for each SNP-probe pair discovered, shows that the discoveries are most concentrated closest to the TSS (at left in Fig 2). Among the discovered distal eSNPs, 50% also appear to act as local regulators, a phenomenon that has been noted before [27–28]. On average, distal eSNPs affect 2.0 probes (median = 1.0), or 1.8 genes; the distribution of the number of genes controlled by distal eSNPs is shown at center in Fig 2. Utilizing the annotations from the Epigenomics Roadmap, we found that 27% of distal eSNPs fall within narrow peaks (which reflect point sources such as transcription factors or chromatin marks associated with transcription start sites) and 38% fall within broad domains (which cover extended areas associated with many other types of histone modifications), indicating that a substantial portion of distal eSNPs are located within functional genomic regions. The most strongly associated local eSNP to each probe with local associations had an average effect size (absolute value of the estimated regression coefficient) of magnitude 0.12; the comparable average for the distal setting was 0.21. The distributions of effect sizes for local and distal regulation are shown at right in Fig 2: the appreciable difference in effect sizes is likely due to the “winner’s curse” phenomenon given the large number of distal hypotheses. Specifically, due to the more stringent selection criteria in the distal setting, there is effectively a higher threshold on the estimated effect sizes for distal associations, so larger eSNP effect sizes are to be expected and may not necessarily reflect a biological difference between distal and local regulation. One possible way to explore the effect of this bias would be to compute false coverage rate confidence intervals for each estimated coefficients (where wider intervals reflect stronger selection bias); this is not completely straightforward given the hierarchical selection procedure, but is of interest in future work. A more detailed investigation of the percentage of variance explained by local and distal eSNPs is given in a later section. For a comparison of the number of discoveries under different error controlling strategies and their characteristics, see S1 Table and S3 Fig.

### Cross-study comparison of discovered eSNPs

To compare our discovered local eSNPs with those of other studies, we rely on the named genes they appear to regulate. This allows us to implicitly account both for the effect of linkage disequilibrium and the different genotypes available. Considering first local association and matching on gene name, our study and published studies had 14,174 gene names in common; 6,456 have significant local associations in our work, and 7,755 have local associations with p<0.0001 in the published studies. Of the 6,456 genes we find significant and on which we have available data in other studies, 4,790 are significant in other studies (430 are significant in one other study, 1,354 are significant in two other studies, 1,182 are significant in 3 other studies and 1,194 are significant in all 4 studies examined).

Examination of distal associations in our work and published studies indicates 10,002 gene names in common; 409 have significant distal associations in our work, and 528 genes have distal associations with p<5e-08 in the published studies. Of the 409 genes we find significant and on which we have data available in other studies, 63 are significant in other studies (17 in one other study, 24 in two studies, 16 in three studies, and 6 in all four studies examined). Only 34 of these 63 genes identified as being significantly affected by distal variants in our study were also identified as having significant distal associations in published work to SNPs on the same chromosome as ours; and 23 of these 34 genes involved associations to SNPs <2Mb apart in our study compared to the published studies (S2 Table).

We examined whether the same SNPs were involved in distal associations in multiple studies, without specifying that the associations were to the same genes. We considered this question matching both on SNP name and on SNP position, requiring that the SNPs were selected as eSNPs in our work at FDR 5% and had associations in published studies at p<5e-08. We found 33 SNPs on six chromosomes to have distal associations to one or more genes in both our study and in published studies (p<5e-08); however the distal associations were to different genes (S3 Table).

There are only ten distal associations significant in our work (controlling the expected average proportion of false associations involving the selected eSNPs to 5%) and in published studies (p<5e-08) that involve the same SNP and same gene: (1) *LIMS1* on chromosome 2 at ~10.9Mb is associated to five SNPs on chromosome 6, at 32.4–32.7Mb (rs13192471, rs3129934, rs3763313, rs9268877, rs9272219) in our work and in [27]; (2) three probes in *DUSP22* on chromosome 6 at ~0.35Mb are associated to one SNP on chromosome 16 at ~35Mb (rs12447240) and is also associated to this gene in [25]; (3) *OR2AG1* on chromosome 11 at ~6.8Mb is associated to one SNP on chromosome 21 at 34.6Mb (rs1131964) in both our study and [25]; (4) *TSSC4* on chromosome 11 at ~2.4Mb is associated to one SNP on chromosome 6 at ~31.2Mb (rs3131018) in both our study and [27]; (5) *NOMO1* on chromosome 16 at ~14.9Mb is associated to one SNP on chromosome 16 at ~16.3Mb (rs4780600) in both our study and [25] (6) and lastly *RTF1* on chromosome 15 at ~41.7Mb is associated to one SNP on chromosome 17 at 2.5Mb (rs8081803) in both our work and [25].

The sparser concordance of the inferred distal vs. local regulation in the cross-study comparison is not surprising: the power to detect distal effects is considerably smaller in all studies, while the impact of confounders stronger. Two additional reasons might explain this difference. On the one hand, our methodology to identify distal eSNPs has larger power to discover multiple genes regulated by the same variant. On the other hand, some of our unique findings might be due to the ascertainment of the subjects, who are members of families carrying genes predisposing to BP1 and/or to extreme values of BP1-related quantitative traits.

### Proportion of heritability explained by eSNPs

**Fig 3:**
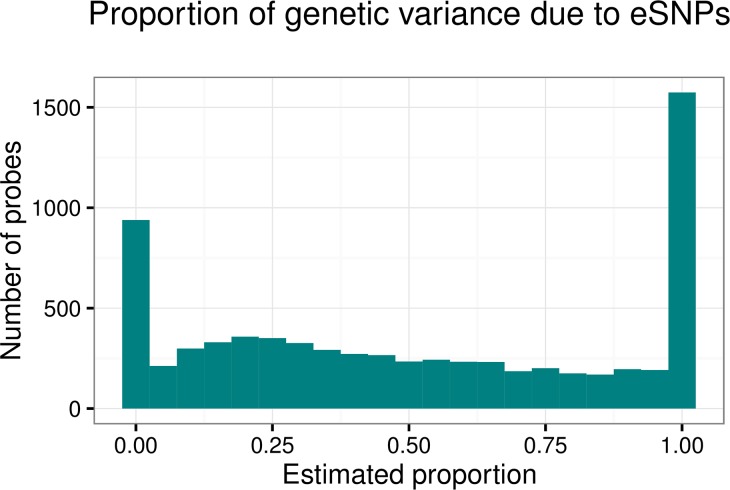
Distribution of proportion of genetic variance due to eSNPs. Proportion of genetic variance due to eSNPs (estimated as 1—the ratio of the genetic variance component when eSNPs are included as fixed effects to the genetic variance component when eSNPs are not included) for the 7,280 heritable probes with local or distal associations, assuming values less than 0 (12%) are exactly 0.

To examine the explanatory power of the discovered eSNPs, we focus on the probes that they affect. Of the 10,065 probes associated to any eSNPs, 7,280 were significantly heritable at an FDR of 5% (7,000 with local associations, 916 with distal associations, and 636 with both). Among the non-heritable probes with eSNP associations, 94% had only local associations, suggesting that these discoveries reflect the less stringent multiplicity control for the discovery of local associations. When the eSNPs for each of the 7,280 heritable probes were included as fixed effects in a variance components model of the probe expression, the genetic variance component was estimated to be 0 for 1,491 (20%) of the probes, indicating that for these probes, the eSNPs capture essentially all of the genetic component of variation in probe expression. The distribution of the proportion of genetic variance due to the selected eSNPs (estimated as 1—the ratio of the genetic variance component when eSNPs are included as fixed effects to the genetic variance component when eSNPs are not included) is shown in Fig 3, assuming values less than 0 (12%) are exactly 0. The median proportion of variance explained for the set of probes with only local, only distal, or both types of associations is 0.44, 0.52, and 0.97, respectively, demonstrating that the eSNPs do explain a substantial proportion of the heritability of gene expression, particularly for probes with both significant local and significant distal associations. The distributions of the local and total genetic proportions of variance under partitioning using GCTA for probes with only local, only distal, or both types of associations (S4 Fig) demonstrates that probes with local associations do in fact have larger proportions of variance due to local effects vs. probes not associated to any local SNPs.

To understand the number of independent signals represented by the eSNPs, we obtained the results from model selection using eSNPs as the pool of possible predictors, focusing again on the set of 7,280 significantly heritable probes with associations to any eSNPs. For this set, the median number of eSNPs with significant marginal association was 23 (mean 41.5), with 521 probes (7.2%) associated to only one eSNP. The distribution of the number of eSNPs included in the best multivariate linear model had a median of 2 (mean 3.1), with 5,328 probes (73%) associated to multiple eSNPs. The large discrepancy in the number of associated SNPs underscores the fact that a substantial proportion of the pairwise SNP-probe associations is due to linkage disequilibrium among neighboring SNPs. At the same time, it is interesting that the selected linear model includes multiple SNPs for 73% of the probes considered: this observation can be interpreted as the result of multiple variants with regulatory effects, but also as a sign that the causal variant is not typed and multiple typed SNPs allow a better reconstruction of the associated haplotype. It is also possible, however, that probes with multiple regulatory SNPs are more likely to appear in the set of significantly heritable probes with any eSNP associations vs. those regulated by a single SNP.

**Fig 4:**
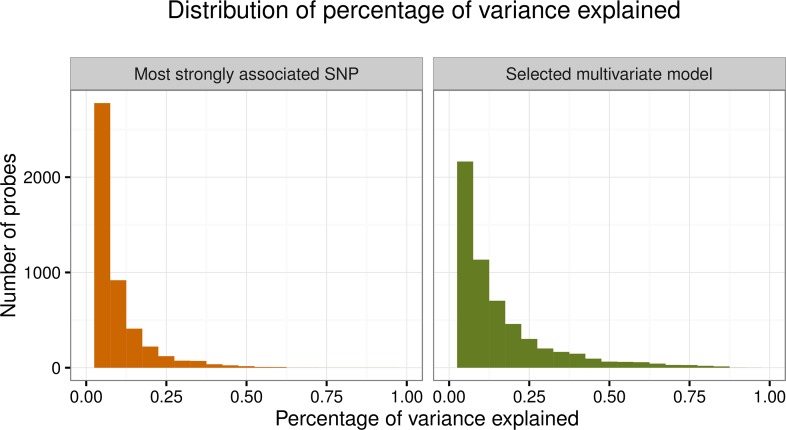
Distribution of percentage of variance explained. Percentage of variance explained (model r^2^) by the most strongly associated eSNP and by the best set of eSNPs selected using multivariate model selection for the 7,280 heritable probes with local or distal eAssociations.

To gain insight into the explanatory power of the univariate vs. multivariate models, we assessed the percentage of total phenotypic variance explained by the most significantly associated SNP and by the selected multivariate linear model. The distribution of the percentage of variance explained for the most significantly associated SNP (Fig 4) has a median of 3.6%, around half that of the results from [25] (median = 7.7%). The median value of r^2^ increases from 3.6% in the univariate model to 7.1% for the best multivariate model (Fig 4). To understand how much heritability was captured by the linear models involving the eSNPs, we also computed the ratio of the percentage of variance explained for the univariate and multivariate models to the probe heritability estimated using the variance components model. This ratio has median 15% for most significantly associated SNP and 29% for the best multivariate model.

## Discussion

The eQTL study of LCL expression in subjects from extended families segregating for BP1 allows us to tackle questions of general interest as well as possibly identifying regulatory variants specific to this sample. Taking advantage of the pedigree information, we can provide estimates of heritability of the expression traits, as well as compare the results of different estimating procedures, relying on theoretical kinship coefficients or on empirical correlations between observed genotypes. Our results suggest that variation in expression values is heritable and that, at least in samples including related individuals, relying on theoretical kinship coefficients or on realized genotype correlation for estimation of heritability leads to similar results. Although we found no association between gene expression and bipolar disorder (BP1) in our study population, it is important to keep in mind that the subjects were recruited as members of extended pedigrees with a history of bipolar disorder, and therefore do not represent a random sample of the general population; the sample ascertainment could impact our findings, particularly with respect to the discovery of SNP-gene associations where the SNP and/or gene are relevant to BP1.

Previous studies have obtained a wide range of estimates of the heritability of gene expression, likely due at least in part to the variety of designs that they have employed and tissues evaluated. Our heritability estimates (18% of probes had heritability > 0.2) are larger than those reported by [29], who found, in a study of LCL expression in trios from different populations, that 10% and 13% out of 47,294 probes had heritability > 0.2 in Europeans and Yorubans, respectively. In a study of peripheral blood expression in 654 complete twin pairs (1,308 subjects), [26] report 4.2% of 18.4K genes to be significantly heritable at FDR<5%, and mean heritability of these significantly heritable probes was 0.15.

On the other hand, our heritability estimates are much smaller than those of [30], who studied lymphocyte expression in large extended families (1,240 subjects in 30 families). They found that 85% of 19,648 probes were significantly heritable at FDR<5%, and median heritability over all probes was 0.23. Grundberg et al. [9], who studied 856 female twin pairs, obtained similarly high values, estimating that the average heritability of expression in LCL of 23,596 probes is 0.21. Considering only the 17% of probes with cis eQTL at FDR<1%, they found average heritability to be 0.25. One reason for the higher estimates of heritability obtained in [9] and [30] is their more stringent filtering of probes based on detection; we observed in our data set that the number of subjects in which a probe is detected is highly correlated with mean expression and positively associated with estimated probe heritability (see S5 Fig).

We found that the strategy used for normalization of gene expression had a large impact on the final heritability estimates (S1 Text and S6 Fig). Specifically, we observed that normalization of gene expression within pedigrees (following the method described in [31]) inflated estimates of heritability substantially over those obtained using global normalization across all subjects, resulting in values more comparable to those of [9] and [30]. Given that the expression levels of individual genes might be expected to differ across pedigrees, but that global differences are likely due to technical or batch effects, we concluded that the heritability values obtained using within-pedigree normalization were artificially high.

Variance decomposition approaches suggest that on average 30% of the genetic variance is due to local regulation. In the majority of probes under local regulation in our sample, more than one typed SNP is required to account for expression variation. This finding can be interpreted as the result of heterogeneity, but also could reflect un-typed causal variants that are tracked by more than one typed SNP.

In the effort to control the rate of false discovery among actually reported results (SNPs with apparent regulatory effects), we adopted a hierarchical multiple comparison controlling procedure that is specifically targeted to eQTL studies. It takes into account differences in local and distal regulation, the likelihood that a variant with distal effects might influence the expression of multiple probes, and the dependence between tests for association involving neighboring SNPs. Our major finding is the identification of eSNPs: variants that regulate gene expression. Our results compare favorably with those of more traditional approaches controlling FDR for the entire collection of SNP-probe associations (i.e. the Benjamini-Hochberg procedure applied across the entire collection of hypotheses): as shown in S3 Fig, our local eSNPs are closer to the TSS, and our distal eSNPs regulate more genes. Although the hierarchical procedure does mildly encourage the association of one SNP to multiple genes, the observed increase in the number of genes regulated by distal SNPs is potentially meaningful, particularly in light of the fact that one of the most common biological explanations for distal effects is that the distal region is controlling a transcription factor, which by definition has an effect on the expression of multiple genes.

A question of general interest is how the list of eSNPs we have obtained relates to the genetic underpinnings of the numerous phenotypes available in these pedigrees. Given that the architecture of these traits is more complex than gene expression, and given our limited sample size, gene mapping is more successful for these traits when relying on a linkage rather than association. The lack of a substantial number of significant SNP associations for these traits makes it impossible to evaluate if eSNPs are enriched in this group. Linkage regions, on the other hand, are wide enough that contrasting the percentage of eSNPs within them and outside them is also rather uninformative. The knowledge we acquired by studying the genetic regulatory network within these pedigrees, instead, can be used to inform our mapping studies: eSNPs might receive a higher prior probability of association, or be assigned a larger portion of the allowed global error rate when using a weighted approach to testing. We will report elsewhere on the results of these investigations.

## Supporting Information

S1 Text Supplementary Text. Detailed information on gene expression and genotype sample collection, processing, and quality control, subject screening procedures, and association of gene expression to BP1.
(PDF)

S1 Fig Comparison of gene expression heritability estimates. Scatterplot of kinship-based heritability estimates obtained using Mendel vs. estimates of the proportion of phenotypic variability explained by genome-wide SNPs obtained using GCTA for all 34,030 probes (upper left). Scatterplot of GCTA estimates for the proportion of phenotypic variability explained by genome-wide SNPs constrained to the range 0 to 1 vs. unconstrained estimates (upper right). Scatterplot of the estimates of probe heritability obtained using a linear mixed model with additive and environmental components only vs. those when an additional family variance component is included (lower left). Scatterplot of the estimates of probe heritability obtained using Mendel with a family variance component included vs. estimates of the proportion of phenotypic variability explained by genome-wide SNPs obtained using GCTA (lower right).
(PDF)

S2 Fig QQ plots. QQ plots for local vs. distal association p-values obtained using Mendel for a randomly chosen probe and for all 34,030 probes genomewide. Due to the large number of tests, in the genomewide setting only p-values for local association < 0.05 for distal association < 0.001 were saved. For the local and distal association analyses across all probes, 7 and 13 p-values respectively were recorded as exactly 0 due to limited precision in Mendel; these are omitted from the plots. The QQ plot for local association for a specific probe (upper left) shows enrichment of small p-values vs. what would be expected under the null; this deviation makes sense, however, given that most genes are subject to some form of local regulation. The QQ plot for distal association for a specific probe (upper right) shows that the distribution of test statistics is as expected under the null. The genomewide distributions (lower left and right) suggest that there are a large number of non-null hypotheses for both local and distal regulation; the deviation from expected values takes place much earlier in the local regulation plot, however, suggesting that the proportion of non-null hypotheses is indeed higher among local vs. distal hypotheses.
(PDF)

S3 Fig Characteristics of local and distal eSNPs. Position of local eSNPs relative to transcription start site (TSS) of the gene queried by the associated probe (left). Number of genes controlled by distal eSNPs (right), excluding SNP kgp22834062, which was associated to more than 95 genes under all methods. Methods compared include Benjamini-Hochberg (BH), hierarchical Benjamini-Hochberg (HBH) and hierarchical Benjamini-Yekutieli (HBY). On average, the distal eSNPs discovered under BH, HBH and HBY are associated to 1.4, 1.5 and 1.8 genes, respectively.
(PDF)

S4 Fig Local and total genetic proportions of variance. Local and total genetic proportions of variance under partitioning using GCTA for the 7,280 heritable probes (FDR<0.05) with local or distal eAssociations.
(PDF)

S5 Fig Probe detection. Scatterplots showing the relationship between the number of subjects in which a probe was detected and mean expression (top), estimated heritability (lower left), and the number of local associations discovered (lower right). Although mean expression, estimated heritability, and the number of local associations are generally increasing with the number of subjects in which the probe was detected, values at the lower end fit reasonably into the overall continuum.
(PDF)

S6 Fig Comparison of heritability estimates under different normalization schemes. Scatterplot showing the relationship between heritability estimates obtained across all probes when expression levels were normalized either within pedigrees or across all subjects. Normalization within pedigree results in inflated heritability estimates (median = 0.20) vs. global normalization (median = 0.03).
(PDF)

S1 Table Number of discoveries under different error control methods. Columns for eSNPs, probes and associations correspond to number of unique SNPs, probes and SNP-probe associations which were significant under the given method. Methods include the Benjamini-Hochberg procedure (BH) across the full set of SNP-probe association hypotheses, as well as two versions of hierarchical error control: hierarchical Benjamini-Hochberg (HBH) and hierarchical Benjamini-Yekutieli (HBY). Under HBH, we apply the BH procedure in the first stage (to discover eSNPs) and the BB procedure in the second stage (to discover their associations), while under HBY, we apply the Benjamini-Yekutieli procedure in the first stage, and the BB procedure in the second stage. All methods are applied targeting level 0.05.
(PDF)

S2 Table Distal associations found in previous studies. Distal associations that were significant in our work (controlling the expected average proportion of false SNP-probe association discoveries involving the selected eSNPs to 5%) and in published studies (p<5e-08) that involve the same gene and SNPs on the same chromosome within 2Mb of our eSNP. Results for the most significant SNP are presented.
(PDF)

S3 Table Distal eSNPs found in previous studies. SNPs with distal association to one or more genes in our work (FDR 5%) and with distal association in published studies to any gene (p<5e-08). The SNP positions are based on hg 19. "# genes" denotes the number of genes in the current study, while "# genes comp" denotes the number of genes discovered in the comparison studies.
(PDF)
