# Supplementary material for: Characterization of Expression Quantitative Trait Loci in Pedigrees from Colombia and Costa Rica Ascertained for Bipolar Disorder
Source: PLoS Genet. 2016 May 13;12(5):e1006046. doi: 10.1371/journal.pgen.1006046 (PMC4866754; doi:10.1371/journal.pgen.1006046)
Supplement: S1 Text — Detailed information on gene expression and genotype sample collection, processing, and quality control, subject screening procedures, and association of gene expression to BP1. (PDF) [file pgen.1006046.s001.pdf]

Supplementary Text.

Characterization of expression quantitative trait loci in pedigrees from Colombia and Costa Rica ascertained for bipolar disorder. C. B. Peterson, S. K. Service, A. J. Jasinska, F. Gao, I. Zelaya, T. M. Teshiba, C. E. Bearden, R. M. Cantor, V. I. Reus, G. Macaya, C. López-Jaramillo, M. Bogomolov, Y. Benjamini, E. Eskin, G. Coppola, N. B. Freimer, and C. Sabatti.

RNA Extraction and Measurement of Gene Expression

Lymphoblastoid cell lines (LCLs) were established at two sites: RUCDR Infinite Biologics (n=549) and UCLA (n=237); RNA was extracted from the cell lines at both sites. Peripheral blood mononuclear cells (PBMCs) were separated from venous blood (which had been preserved with an anticoagulating reagent, typically ACD), using either Nycoprep (RUCDR) or Ficoll-Paque PLUS (UCLA) as the separation medium. Both sites used a standard protocol to transform freshly separated or frozen PBMCs, employing media containing Epstein Barr virus (EBV) and the mitogen phytohemagglutinin (PHA). After the culture became established, both sites pelleted cells by centrifugation at 300g for 10 minutes and immediately lyzed them with RLT buffer containing beta-mercaptoethanol, mixed them briefly, and stored them at -80° C until the RNA extraction.

Both RUCDR and UCLA extracted RNA from the cultured cells with an RNeasy 96 kit (Qiagen, Venlo, Netherlands), employing either a Qiagen BioRobot Universal System (RUCDR) or a manual procedure (UCLA) using up to $5x10^5$ cells (RUCDR) or 10^6 cells (UCLA) as the starting material. To quantify RNA yield, we used a Quant-iT RiboGreen (Invitrogen, Waltham, MA) and measured RNA integrity number, reflective of sample quality, using a TapeStation (Agilent, Santa Clara, CA).

To evaluate gene expression we amplified total RNA (100ng), labeled it using Ambion Total Prep-96 kits (Life Technologies, Grand Island, NY), and hybridized it on Illumina Human HT-12 v4.0 Expression BeadChips (Illumina Inc., San Diego, CA). Arrays were scanned with an Illumina iScan confocal instrument. Each chip queries the expression of approximately 47,000

probes relative to 31,223 gene targets, as defined by the NCBI reference sequence (RefSeq) database. For these experiments we processed the 786 samples in nine batches (eight batches of between 90-95 samples and one batch of 44 samples).

Data analysis was performed using R (www.r-project.org) and Bioconductor (www.bioconductor.org) packages. Expression values were background corrected, quantile normalized, and log2 transformed using the function necq [1]. We corrected for the major known batch effects (RUCDR vs. UCLA LCL construction and RNA extraction) using ComBat [2], including BP1 diagnosis, sex, and pedigree IDs as covariates.

The decision to normalize gene expression across all subjects rather than within pedigree (as proposed in [3]) strongly influenced our estimates of expression heritability. We chose to normalize across all subjects, rather than within pedigrees, out of the concern that the much higher heritability estimates (median=0.20) obtained using within-pedigree normalization could reflect what is essentially the introduction of an artificial batch effect (S6 Fig). Given that the goal of normalization is to eliminate artifacts, and that the expression of individual genes might reasonably be expected to differ across families but not global expression levels, we choose to normalize across all subjects. Moreover, we found that the PEER factors (latent factors explaining global gene expression) [4] were not strongly correlated with pedigree membership, suggesting that family effects do not make a major contribution to global gene expression differences.

Gene expression quality control

We filtered out 12,834 probes from the initial set of 47,009 probes because of not aligning to hg19 (n=522), aligning to > 2 locations (1,622), having one or more mismatches in the probe sequence (1,509), or spanning across one or more SNPs in dbSNP 137 or 138 (6,040). While some authors adopt stringent filtering based on detection, retaining only probes with $p < 0.05$ in at least 50% of samples [5] or at least 5% of samples [6-7] others do not filter on the basis of detection at all [8-9]. S5 Fig shows the relationship between the number of subjects in which a probe was detected and mean expression, estimated heritability and the number of local associations; given that the probes detected in few samples fit into the overall continuum, we

opt for a reasonably generous threshold of inclusion, filtering out 3,141 probes because they were not detected in at least one sample with an Illumina detection threshold <0.05. After these probes were removed, there were 99 genes interrogated by more than 1 probe where at least one of the probes involved mapped to different locations. Of the mismatching probes, the set with multiple alignments were filtered out (114). In addition, all probes were filtered out for the 12 genes where the probes that map to different chromosomes did not have multiple alignments (31). After these quality control steps, 34,030 probes remained for analysis.

Genotype quality control and filtering

Genotype data were obtained for 856 subjects. A total of 2,026,257 SNPs were polymorphic and passed all QC procedures. SNPs were excluded in the QC process due to discordance among the three batches in replicated individuals (8,280 SNPs), missing >5% of data (97,158 SNPs), gross violation of Hardy-Weinberg equilibrium (HWE, 79 SNPs), and presence of >4 Mendel errors among fully typed trios (2,976 SNPs). After excluding markers with >4 Mendel errors, the Mendel error rate among fully typed trios was 0.01%, and all further sporadic errors were set to missing in the entire trio. All allele frequency calculations, calculations of HWE, and estimates of LD were performed using only unrelated (founder) individuals. Association analyses used 1,024,051 autosomal SNPs with MAF>10%.

Screening of subjects

Eighteen subjects were excluded from final analysis because of sample mix-up/contamination (12 subjects) or because they were married-in individuals whose children were not recruited into the study (6 subjects). Among the 838 subjects that passed SNP QC, genotyping completeness was good, averaging 99.78%. Only one subject had genotyping completeness <95%; as they were BP1 and they were missing only 5.7% of genotypes, we retained them for analysis. There were 786 individuals with both genotype and gene expression data (193 BP1 and 593 non-BP1).

Relationship between gene expression levels and BP1 diagnosis

We focus here on two categories of related individuals: those with a clinical diagnosis of BP1 vs. those with no history of BP1. We would like to note that the subjects with no history of BP1 are not necessarily healthy controls; they are included in the sample because they are related to a BP1 subject and may have a range of other non-BP1 diagnoses. To identify genes with differential expression in the BP1 vs. non-BP1 individuals accounting for the correlation induced by relatedness, we used a variance components approach, as implemented in Mendel [10]. Specifically, we computed p-values for the association of BP1 to gene expression by fitting a variance components model with additive genetic and environmental components and BP1 status as predictor, as well as additional covariates corresponding to age, sex, batch and PEER factors. To correct for the multiplicity of tests, we applied the Benjamini-Hochberg (BH) procedure [11] to control the false discovery rate (FDR) to the 5% level. We did not detect statistically significant differences between the residual mean expression for BP1 subjects (n=193) and their non-BP1 relatives (n=593) after correcting for multiple comparisons. One probe had $p<5e-05$: ILMN_1805371 on chromosome 19 at 8.5Mb (querying the expression of ARMCX3 on chromosome X, p=1.65e-05). However, no comparisons were significant at an FDR threshold of 5%. Since BP1 status was not associated with differences in gene expression, we did not explicitly adjust for BP1 status in the remaining analysis. We would like to note that while BP1 status was not associated to gene expression for any genes in the current study, the fact that subjects were recruited for a family history of BP1 may limit the generalizability of our findings to subjects with no family history of BP1.

References

1. Shi W, Oshlack A, Smyth GK. Optimizing the noise versus bias trade-off for Illumina Whole Genome Expression BeadChips. Nucleic Acids Res. 2010;38(22):e204.

2. Johnson WE, Li C, Rabinovic A. Adjusting batch effects in microarray expression data using empirical Bayes methods. Biostatistics. 2007;8(1):118–127.

3. Kim Y, Doan BQ, Duggal P, Bailey-Wilson JE. Normalization of microarray expression data using within-pedigree pool and its effect on linkage analysis. BMC Proc. 2007;1 Suppl I:S152.

4. Stegle O, Parts L, Piipari M, Winn J, Durbin R. Using probabilistic estimation of expression residuals (PEER) to obtain increased power and interpretability of gene expression analyses.

Nat Protoc. 2012;7(3):500–507.

5. Powell JE, Henders AK, McRae AF, Wright MJ, Martin NG, Dermitzakis ET, et al. Genetic control of gene expression in whole blood and lymphoblastoid cell lines is largely independent. Genome Res. 2012;22(3):456–466.

6. Zeller T, Wild P, Szymczak S, Rotival M, Schillert A, Castagne R, et al. Genetics and beyond–the transcriptome of human monocytes and disease susceptibility. PloS One. 2010;5(5):e10693.

7. Price AL, Helgason A, Thorleifsson G, McCarroll SA, Kong A, Stefansson K. Single-tissue and cross-tissue heritability of gene expression via identity-by-descent in related or unrelated individuals. PloS Genet. 2011;7(2):e1001317.

8. Fairfax BP, Makino S, Radhakrishnan J, Plant K, Leslie S, Dilthey A, et al. Genetics of gene expression in primary immune cells identifies cell type-specific master regulators and roles of HLA alleles. Nat Genet. 2012;44(5):502-511.

9. Westra H, Peters MJ, Esko T, Yaghootkar H, Schurmann C, Kettunen J, et al. Systematic identification of trans eQTLs as putative drivers of known disease associations. Nat Genet. 2013;45(10):1238–1243.

10. Lange K, Papp JC, Sinsheimer JS, Sripracha R, Zhou H, Sobel ES. Mendel: the Swiss army knife of genetic analysis programs. Bioinform. 2013;29(12):1568–1570.

11. Benjamini Y, Hochberg Y. Controlling the false discovery rate: a practical and powerful approach to multiple testing. J R Stat Soc Series B Methodol. 1995;57(1):289–300.
