# Supplementary material for: Characterization of Expression Quantitative Trait Loci in Pedigrees from Colombia and Costa Rica Ascertained for Bipolar Disorder
Source: PLoS Genet. 2016 May 13;12(5):e1006046. doi: 10.1371/journal.pgen.1006046 (PMC4866754; doi:10.1371/journal.pgen.1006046)
Supplement: S1 Fig — Scatterplot of kinship-based heritability estimates obtained using Mendel vs. estimates of the proportion of phenotypic variability explained by genome-wide SNPs obtained using GCTA for all 34,030 probes (upper left). Scatterplot of GCTA estimates for the proportion of phenotypic variability explained by genome-wide SNPs constrained to the range 0 to 1 vs. unconstrained estimates (upper right). Scatterplot of the estimates of probe heritability obtained using a linear mixed model with additive and environmental components only vs. those when an additional family variance component is included (lower left). Scatterplot of the estimates of probe heritability obtained using Mendel with a family variance component included vs. estimates of the proportion of phenotypic variability explained by genome-wide SNPs obtained using GCTA (lower right). (PDF) [file pgen.1006046.s002.pdf]

Supporting Information.

Characterization of expression quantitative trait loci in pedigrees from Colombia and Costa Rica ascertained for bipolar disorder. C. B. Peterson, S. K. Service, A. J. Jasinska, F. Gao, I. Zelaya, T. M. Teshiba, C. E. Bearden, R. M. Cantor, V. I. Reus, G. Macaya, C. López-Jaramillo, M. Bogomolov, Y. Benjamini, E. Eskin, G. Coppola, N. B. Freimer, and C. Sabatti.

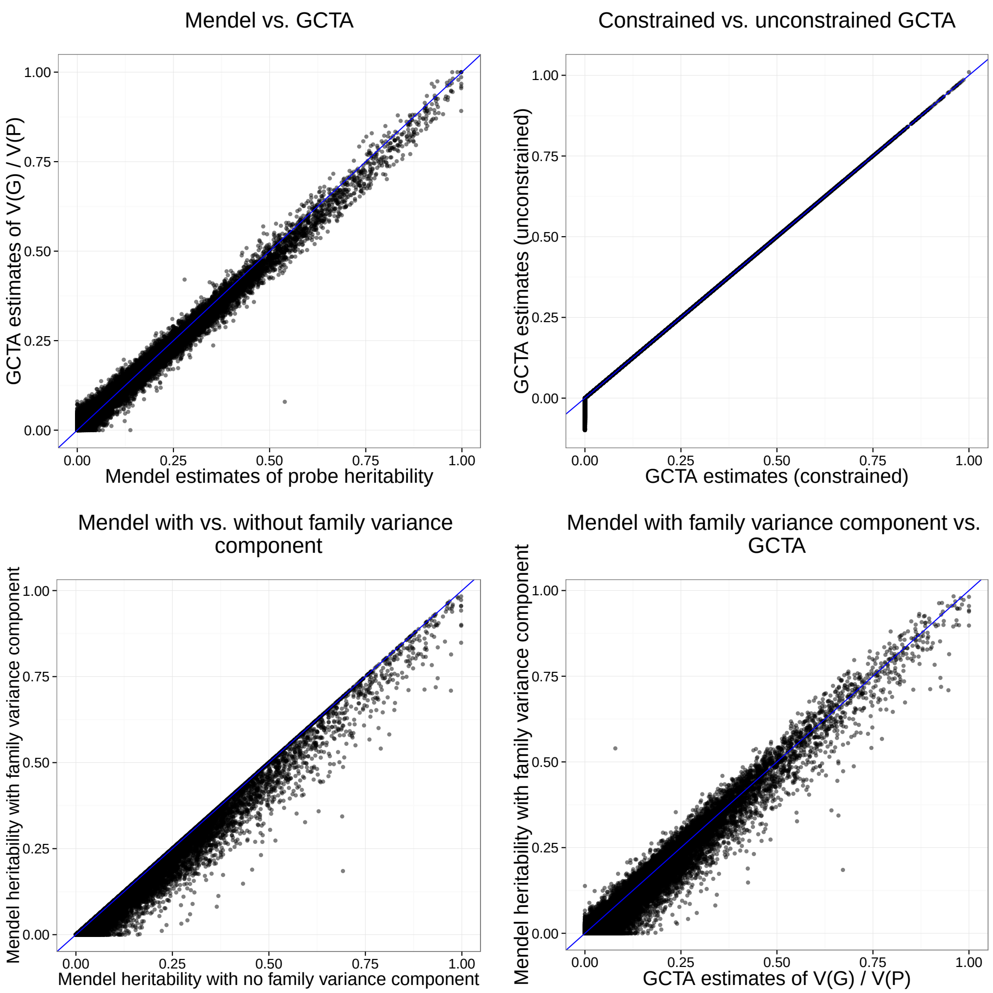

Fig S1. Comparison of gene expression heritability estimates. Scatterplot of kinship-based heritability estimates obtained using Mendel vs. estimates of the proportion of phenotypic variability explained by genome-wide SNPs obtained using GCTA for all 34,030 probes (upper left). Scatterplot of GCTA estimates for the proportion of phenotypic variability explained by genome-wide SNPs constrained to the range 0 to 1 vs. unconstrained estimates (upper right). Scatterplot of the estimates of probe heritability obtained using a linear mixed model with additive and environmental components only vs. those when an additional family variance component is included (lower left). Scatterplot of the estimates of probe heritability obtained using Mendel with a family variance component included vs. estimates of the proportion of phenotypic variability explained by genome-wide SNPs obtained using GCTA (lower right).
