# Supplementary material for: Characterization of Expression Quantitative Trait Loci in Pedigrees from Colombia and Costa Rica Ascertained for Bipolar Disorder
Source: PLoS Genet. 2016 May 13;12(5):e1006046. doi: 10.1371/journal.pgen.1006046 (PMC4866754; doi:10.1371/journal.pgen.1006046)
Supplement: S2 Fig — QQ plots for local vs. distal association p-values obtained using Mendel for a randomly chosen probe and for all 34,030 probes genomewide. Due to the large number of tests, in the genomewide setting only p-values for local association < 0.05 for distal association < 0.001 were saved. For the local and distal association analyses across all probes, 7 and 13 p-values respectively were recorded as exactly 0 due to limited precision in Mendel; these are omitted from the plots. The QQ plot for local association for a specific probe (upper left) shows enrichment of small p-values vs. what would be expected under the null; this deviation makes sense, however, given that most genes are subject to some form of local regulation. The QQ plot for distal association for a specific probe (upper right) shows that the distribution of test statistics is as expected under the null. The genomewide distributions (lower left and right) suggest that there are a large number of non-null hypotheses for both local and distal regulation; the deviation from expected values takes place much earlier in the local regulation plot, however, suggesting that the proportion of non-null hypotheses is indeed higher among local vs. distal hypotheses. (PDF) [file pgen.1006046.s003.pdf]

Supporting Information.

Characterization of expression quantitative trait loci in pedigrees from Colombia and Costa Rica ascertained for bipolar disorder. C. B. Peterson, S. K. Service, A. J. Jasinska, F. Gao, I. Zelaya, T. M. Teshiba, C. E. Bearden, R. M. Cantor, V. I. Reus, G. Macaya, C. López-Jaramillo, M. Bogomolov, Y. Benjamini, E. Eskin, G. Coppola, N. B. Freimer, and C. Sabatti.

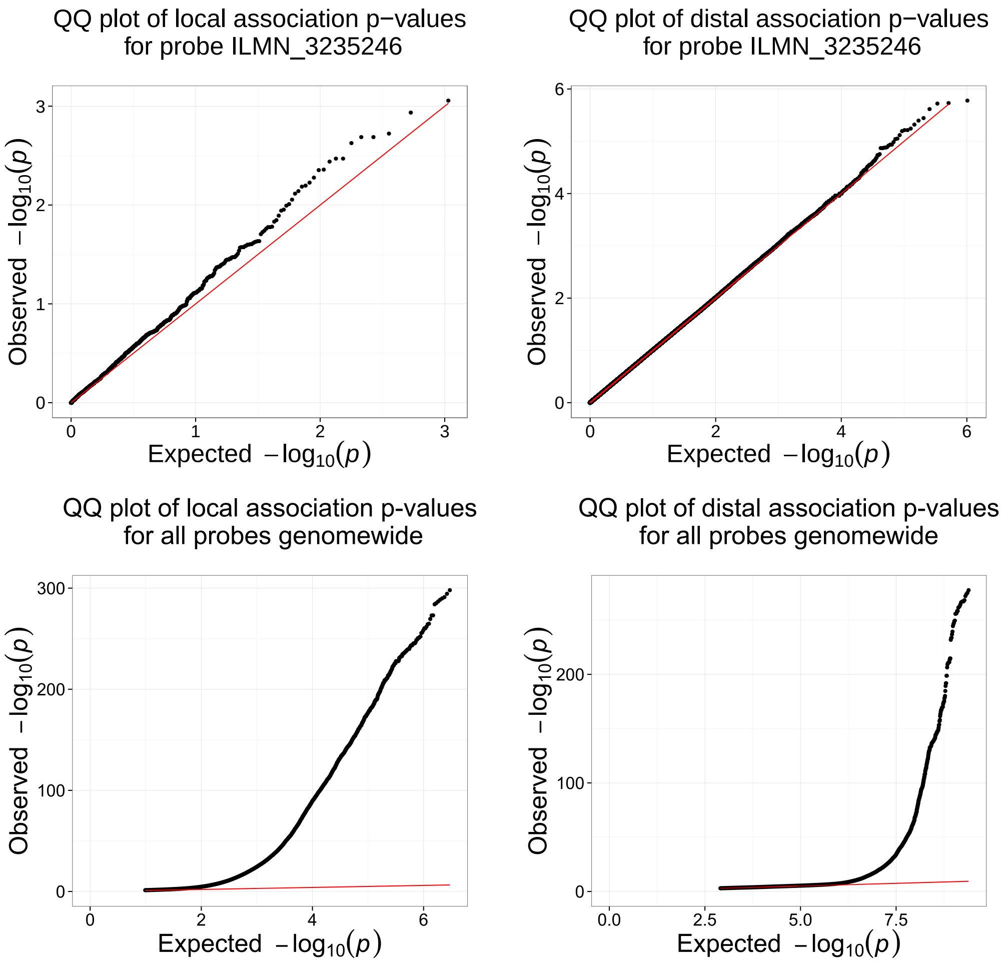

S2 Fig. QQ plots. QQ plots for local vs. distal association p-values obtained using Mendel for a randomly chosen probe and for all 34,030 probes genomewide. Due to the large number of tests, in the genomewide setting only p-values for local association < 0.05 for distal association < 0.001 were saved. For the local and distal association analyses across all probes, 7 and 13 p-values respectively were recorded as exactly 0 due to limited precision in Mendel; these are omitted from the plots. The QQ plot for local association for a specific probe (upper left) shows enrichment of small p-values vs. what would be expected under the null ($\lambda_{GC} = 1.15$); this deviation makes sense, however, given that most genes are subject to some form of local regulation. The QQ plot for distal association for a specific probe (upper right) shows that the distribution of test statistics is as expected under the null ($\lambda_{GC} = 1.01$). The genomewide distributions (lower left and right) suggest that there are a large number of non-null hypotheses for both local and distal regulation; the deviation from expected values takes place much earlier in the local regulation plot, however, suggesting that the proportion of non-null hypotheses is indeed higher among local vs. distal hypotheses.
