# Supplementary material for: Characterization of Expression Quantitative Trait Loci in Pedigrees from Colombia and Costa Rica Ascertained for Bipolar Disorder
Source: PLoS Genet. 2016 May 13;12(5):e1006046. doi: 10.1371/journal.pgen.1006046 (PMC4866754; doi:10.1371/journal.pgen.1006046)
Supplement: S3 Fig — Position of local eSNPs relative to transcription start site (TSS) of the gene queried by the associated probe (left). Number of genes controlled by distal eSNPs (right), excluding SNP kgp22834062, which was associated to more than 95 genes under all methods. Methods compared include Benjamini-Hochberg (BH), hierarchical Benjamini-Hochberg (HBH) and hierarchical Benjamini-Yekutieli (HBY). On average, the distal eSNPs discovered under BH, HBH and HBY are associated to 1.4, 1.5 and 1.8 genes, respectively. (PDF) [file pgen.1006046.s004.pdf]

Supporting Information.

Characterization of expression quantitative trait loci in pedigrees from Colombia and Costa Rica ascertained for bipolar disorder. C. B. Peterson, S. K. Service, A. J. Jasinska, F. Gao, I. Zelaya, T. M. Teshiba, C. E. Bearden, R. M. Cantor, V. I. Reus, G. Macaya, C. López-Jaramillo, M. Bogomolov, Y. Benjamini, E. Eskin, G. Coppola, N. B. Freimer, and C. Sabatti.

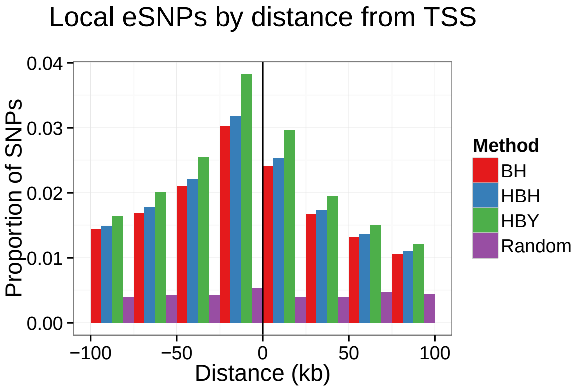

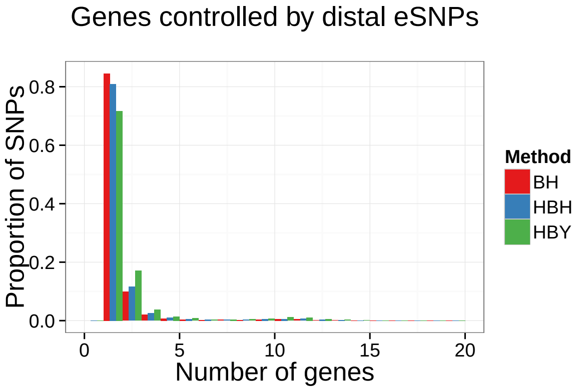

Fig S3. Characteristics of local and distal eSNPs. Position of local eSNPs relative to transcription start site (TSS) of the gene queried by the associated probe (left). Number of genes controlled by distal eSNPs (right), excluding SNP kgp22834062, which was associated to more than 95 genes under all methods. Methods compared include Benjamini-Hochberg (BH), hierarchical Benjamini-Hochberg (HBH) and hierarchical Benjamini-Yekutieli (HBY). On average, the distal eSNPs discovered under BH, HBH and HBY are associated to 1.4, 1.5 and 1.8 genes, respectively.
