# Supplementary material for: Characterization of Expression Quantitative Trait Loci in Pedigrees from Colombia and Costa Rica Ascertained for Bipolar Disorder
Source: PLoS Genet. 2016 May 13;12(5):e1006046. doi: 10.1371/journal.pgen.1006046 (PMC4866754; doi:10.1371/journal.pgen.1006046)
Supplement: S4 Fig — Local and total genetic proportions of variance under partitioning using GCTA for the 7,280 heritable probes (FDR<0.05) with local or distal eAssociations. (PDF) [file pgen.1006046.s005.pdf]

Supporting Information.

Characterization of expression quantitative trait loci in pedigrees from Colombia and Costa Rica ascertained for bipolar disorder. C. B. Peterson, S. K. Service, A. J. Jasinska, F. Gao, I. Zelaya, T. M. Teshiba, C. E. Bearden, R. M. Cantor, V. I. Reus, G. Macaya, C. López-Jaramillo, M. Bogomolov, Y. Benjamini, E. Eskin, G. Coppola, N. B. Freimer, and C. Sabatti.

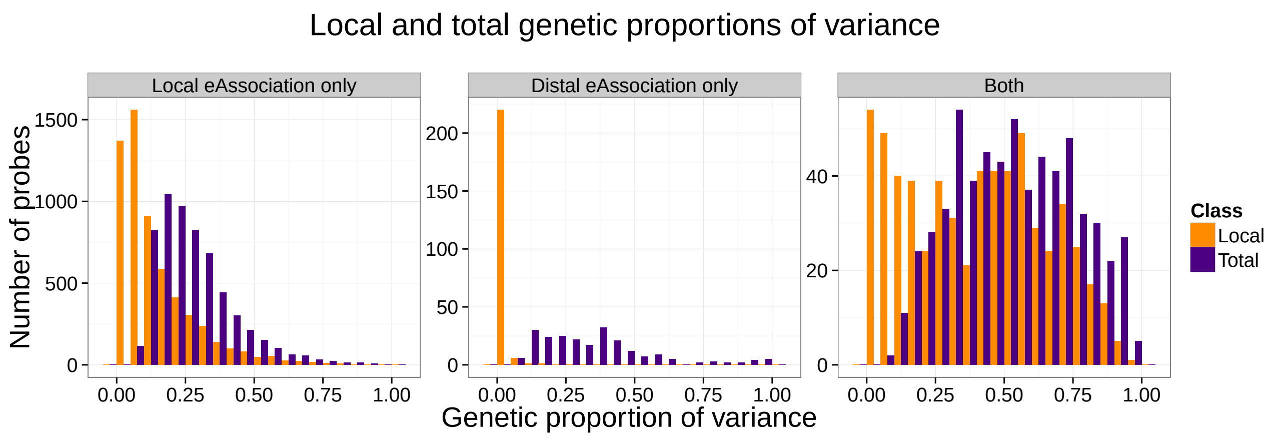

Fig S4. Local and total genetic proportions of variance. Local and total genetic proportions of variance under partitioning using GCTA for the 7,280 heritable probes (FDR<0.05) with local or distal eAssociations.
