# Supplementary material for: Characterization of Expression Quantitative Trait Loci in Pedigrees from Colombia and Costa Rica Ascertained for Bipolar Disorder
Source: PLoS Genet. 2016 May 13;12(5):e1006046. doi: 10.1371/journal.pgen.1006046 (PMC4866754; doi:10.1371/journal.pgen.1006046)
Supplement: S5 Fig — Scatterplots showing the relationship between the number of subjects in which a probe was detected and mean expression (top), estimated heritability (lower left), and the number of local associations discovered (lower right). Although mean expression, estimated heritability, and the number of local associations are generally increasing with the number of subjects in which the probe was detected, values at the lower end fit reasonably into the overall continuum. (PDF) [file pgen.1006046.s006.pdf]

Supporting Information.

Characterization of expression quantitative trait loci in pedigrees from Colombia and Costa Rica ascertained for bipolar disorder. C. B. Peterson, S. K. Service, A. J. Jasinska, F. Gao, I. Zelaya, T. M. Teshiba, C. E. Bearden, R. M. Cantor, V. I. Reus, G. Macaya, C. López-Jaramillo, M. Bogomolov, Y. Benjamini, E. Eskin, G. Coppola, N. B. Freimer, and C. Sabatti.

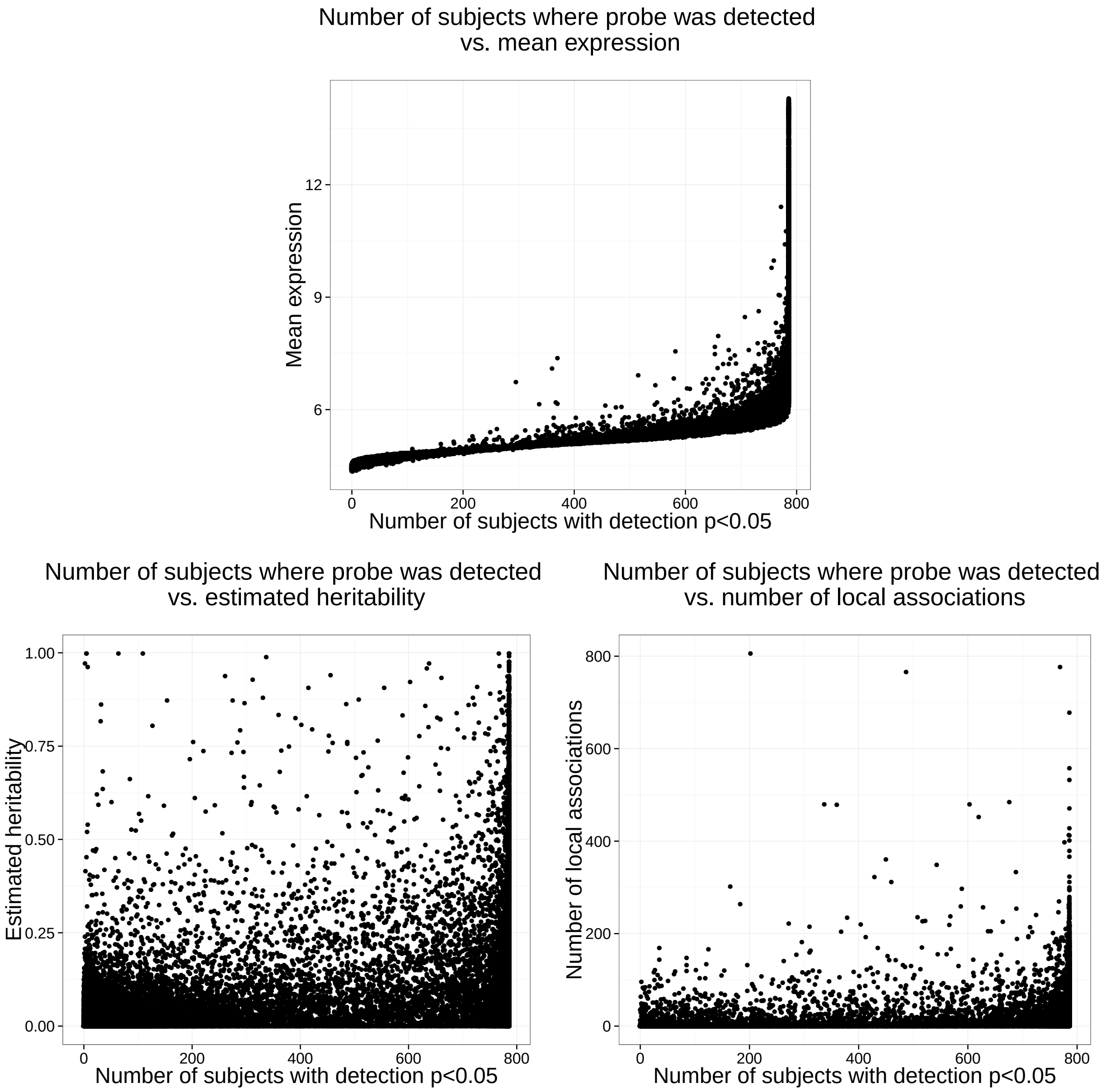

Fig S5. Probe detection. Scatterplots showing the relationship between the number of subjects in which a probe was detected and mean expression (top), estimated heritability (lower left), and the number of local associations discovered (lower right). Although mean expression, estimated heritability, and the number of local associations are generally increasing with the number of subjects in which the probe was detected, values at the lower end fit reasonably into the overall continuum.
