# Supplementary material for: Characterization of Expression Quantitative Trait Loci in Pedigrees from Colombia and Costa Rica Ascertained for Bipolar Disorder
Source: PLoS Genet. 2016 May 13;12(5):e1006046. doi: 10.1371/journal.pgen.1006046 (PMC4866754; doi:10.1371/journal.pgen.1006046)
Supplement: S6 Fig — Scatterplot showing the relationship between heritability estimates obtained across all probes when expression levels were normalized either within pedigrees or across all subjects. Normalization within pedigree results in inflated heritability estimates (median = 0.20) vs. global normalization (median = 0.03). (PDF) [file pgen.1006046.s007.pdf]

Supporting Information.

Characterization of expression quantitative trait loci in pedigrees from Colombia and Costa Rica ascertained for bipolar disorder. C. B. Peterson, S. K. Service, A. J. Jasinska, F. Gao, I. Zelaya, T. M. Teshiba, C. E. Bearden, R. M. Cantor, V. I. Reus, G. Macaya, C. López-Jaramillo, M. Bogomolov, Y. Benjamini, E. Eskin, G. Coppola, N. B. Freimer, and C. Sabatti.

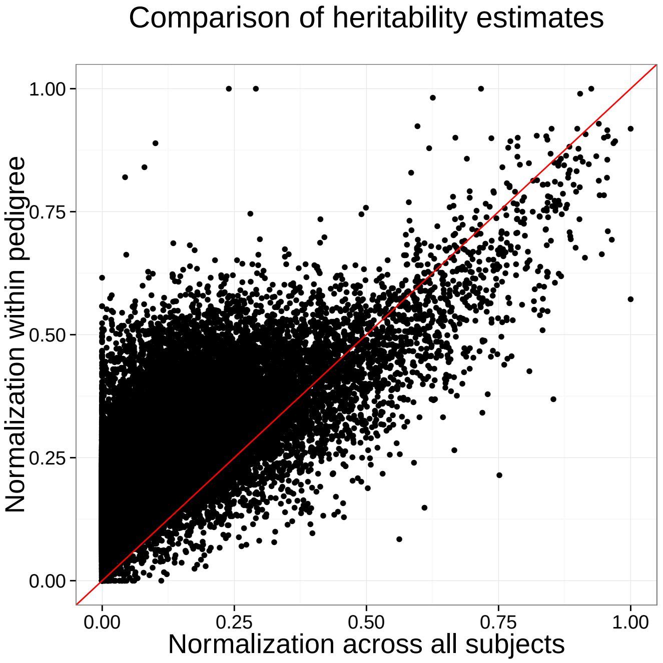

Fig S6. Comparison of heritability estimates under different normalization schemes. Scatterplot showing the relationship between heritability estimates obtained across all probes when expression levels were normalized either within pedigrees or across all subjects. Normalization within pedigree results in inflated heritability estimates (median=0.20) vs. global normalization (median=0.03).
