# Supplementary material for: Characterization of Expression Quantitative Trait Loci in Pedigrees from Colombia and Costa Rica Ascertained for Bipolar Disorder
Source: PLoS Genet. 2016 May 13;12(5):e1006046. doi: 10.1371/journal.pgen.1006046 (PMC4866754; doi:10.1371/journal.pgen.1006046)
Supplement: S1 Table — Columns for eSNPs, probes and associations correspond to number of unique SNPs, probes and SNP-probe associations which were significant under the given method. Methods include the Benjamini-Hochberg procedure (BH) across the full set of SNP-probe association hypotheses, as well as two versions of hierarchical error control: hierarchical Benjamini-Hochberg (HBH) and hierarchical Benjamini-Yekutieli (HBY). Under HBH, we apply the BH procedure in the first stage (to discover eSNPs) and the BB procedure in the second stage (to discover their associations), while under HBY, we apply the Benjamini-Yekutieli procedure in the first stage, and the BB procedure in the second stage. All methods are applied targeting level 0.05. (PDF) [file pgen.1006046.s008.pdf]

Supporting Information.

Characterization of expression quantitative trait loci in pedigrees from Colombia and Costa Rica ascertained for bipolar disorder. C. B. Peterson, S. K. Service, A. J. Jasinska, F. Gao, I. Zelaya, T. M. Teshiba, C. E. Bearden, R. M. Cantor, V. I. Reus, G. Macaya, C. López-Jaramillo, M. Bogomolov, Y. Benjamini, E. Eskin, G. Coppola, N. B. Freimer, and C. Sabatti.

	Method	eSNPs	probes	associations
local	BH	256,395	17,074	475,027
	HBH	220,085	14,550	426,307
	HBY	139,668	9,645	305,635
distal	BH	24,902	4,221	37,450
	HBH	20,912	3,148	35,052
	HBY	11,016	1,081	22,304

Table S1. Number of discoveries under different error control methods. Columns for eSNPs, probes and associations correspond to number of unique SNPs, probes and SNP-probe associations which were significant under the given method. Methods include the Benjamini-Hochberg procedure (BH) across the full set of SNP-probe association hypotheses, as well as two versions of hierarchical error control: hierarchical Benjamini-Hochberg (HBH) and hierarchical Benjamini-Yekutieli (HBY). Under HBH, we apply the BH procedure in the first stage (to discover eSNPs) and the BB procedure in the second stage (to discover their associations), while under HBY, we apply the Benjamini-Yekutieli procedure in the first stage, and the BB procedure in the second stage. All methods are applied targeting level 0.05.
