# Supplementary material for: Characterization of Expression Quantitative Trait Loci in Pedigrees from Colombia and Costa Rica Ascertained for Bipolar Disorder
Source: PLoS Genet. 2016 May 13;12(5):e1006046. doi: 10.1371/journal.pgen.1006046 (PMC4866754; doi:10.1371/journal.pgen.1006046)
Supplement: S2 Table — Distal associations that were significant in our work (controlling the expected average proportion of false SNP-probe association discoveries involving the selected eSNPs to 5%) and in published studies (p<5e-08) that involve the same gene and SNPs on the same chromosome within 2Mb of our eSNP. Results for the most significant SNP are presented. (PDF) [file pgen.1006046.s009.pdf]

Supporting Information.

Characterization of expression quantitative trait loci in pedigrees from Colombia and Costa Rica ascertained for bipolar disorder. C. B. Peterson, S. K. Service, A. J. Jasinska, F. Gao, I. Zelaya, T. M. Teshiba, C. E. Bearden, R. M. Cantor, V. I. Reus, G. Macaya, C. López-Jaramillo, M. Bogomolov, Y. Benjamini, E. Eskin, G. Coppola, N. B. Freimer, and C. Sabatti.

			Our study		Comparison study		
Gene	Gene chr	SNP chr	SNP location	max -log10(p)	Study name	SNP location	max -log10(p)
AVP	chr20	chr4	1203160	11.26	Westra	1355127	32.97
					Zeller	1244415	17.43
BTN3A2	chr6	chr6	29468809	11.82	Westra	32522251	12.40
					Hapmap3	27805254	6.78
					Zeller	27413923	76.36
					Wright	27390115	135.41
CLK3	chr15	chr6	32574603	23.82	Zeller	32429757	8.78
DDX12	chr12	chr12	31225710	9.14	Zeller	31237195	8.37
DEF8	chr16	chr6	32681530	16.92	Westra	32540813	8.68
					Zeller	32580616	39.22
DRD3	chr3	chr16	89976329	44.69	Zeller	89961660	24.25
DUSP22	chr6	chr16	33836510	33.91	Zeller	34928235	23.69
FKBP9L	chr7	chr7	32995331	24.10	Zeller	33003686	147.87
FOXC2	chr16	chr21	45595932	9.01	Zeller	45479054	9.03
HLA-G	chr6	chr6	31582025	14.09	Zeller	28769670	11.08
					Wright	31072723	2.17
LIMS1	chr2	chr6	32577874	86.32	Westra	32712350	46.41
MSRA	chr8	chr8	11382367	12.23	Wright	11521079	7.49
NOMO1	chr16	chr16	16290330	10.80	Zeller	16290494	8.14
OR2AG1	chr11	chr21	34610487	13.60	Zeller	34610486	19.00
PDPR	chr16	chr16	74337036	24.00	Wright	74480417	17.28
					Zeller	74513783	3.92
RTF1	chr15	chr17	2565009	43.15	Zeller	2565008	16.87
SPTBN4	chr19	chr4	1244416	9.74	Zeller	1357324	11.67
					Westra	1355127	13.82
SSRP1	chr11	chr6	32560859	17.74	Zeller	32387808	11.83
					Wright	128215237	1.44
TAP2	chr6	chr6	31392118	11.09	Wright	31740763	7.30
TRIM56	chr7	chr6	32566232	15.22	Westra	32497626	9.57
					Zeller	32431291	4.31
TSSC4	chr11	chr6	29913067	16.51	Westra	30026078	57.20
ZNF514	chr2	chr2	97562551	17.80	Wright	90234525	7.48
					Zeller	96981368	1.95
ZNF672	chr1	chr6	32449411	13.20	Zeller	32429757	5.19
					Westra	32521437	8.60

Table S2. Distal associations found in previous studies. Distal associations that were significant in our work (controlling the expected average proportion of false SNP-probe association discoveries involving the selected eSNPs to 5%) and in published studies (p<5e-08) that involve the same gene and SNPs on the same chromosome within 2Mb of our eSNP. Results for the most significant SNP are presented.
