# Supplementary material for: Characterization of Expression Quantitative Trait Loci in Pedigrees from Colombia and Costa Rica Ascertained for Bipolar Disorder
Source: PLoS Genet. 2016 May 13;12(5):e1006046. doi: 10.1371/journal.pgen.1006046 (PMC4866754; doi:10.1371/journal.pgen.1006046)
Supplement: S3 Table — SNPs with distal association to one or more genes in our work (FDR 5%) and with distal association in published studies to any gene (p<5e-08). The SNP positions are based on hg 19. "# genes" denotes the number of genes in the current study, while "# genes comp" denotes the number of genes discovered in the comparison studies. (PDF) [file pgen.1006046.s010.pdf]

Supporting Information.

Characterization of expression quantitative trait loci in pedigrees from Colombia and Costa Rica ascertained for bipolar disorder. C. B. Peterson, S. K. Service, A. J. Jasinska, F. Gao, I. Zelaya, T. M. Teshiba, C. E. Bearden, R. M. Cantor, V. I. Reus, G. Macaya, C. López-Jaramillo, M. Bogomolov, Y. Benjamini, E. Eskin, G. Coppola, N. B. Freimer, and C. Sabatti.

SNP	SNP chr	SNP position	# genes	# genes comp	# studies
rs10218795	chr1	145416056	1	1	1
rs1732115	chr4	1244416	1	2	1
rs198846	chr6	26107463	1	1	1
rs2860580	chr6	29906691	1	5	1
rs9357092	chr6	29984252	1	2	1
rs3094212	chr6	31085770	2	3	1
rs3131018	chr6	31143582	2	3	2
rs13437082	chr6	31354560	2	2	1
rs9469003	chr6	31407828	1	1	1
rs3094228	chr6	31429927	4	2	1
rs443198	chr6	32190406	4	3	1
rs6910071	chr6	32282854	6	1	1
rs2395163	chr6	32387809	8	3	1
rs6903608	chr6	32428285	8	3	1
rs9268853	chr6	32429643	14	8	2
rs9268877	chr6	32431147	9	3	1
rs2395185	chr6	32433167	14	4	1
rs660895	chr6	32577380	10	3	1
rs9272219	chr6	32602269	12	1	1
rs7774434	chr6	32657578	8	1	1
rs6457617	chr6	32663851	2	2	1
rs2647012	chr6	32664458	3	5	1
rs9357152	chr6	32664960	3	1	1
rs9275390	chr6	32669156	5	1	1
rs9275596	chr6	32681631	1	5	1
rs2069408	chr12	56364321	14	1	1
rs1701704	chr12	56412487	16	7	1
rs11171739	chr12	56470625	18	10	2
rs2292239	chr12	56482180	16	7	1
rs4780600	chr16	16290495	1	2	1
rs12447240	chr16	34928236	1	1	1
rs11659642	chr16	89819932	2	1	1
rs199457	chr17	44795469	7	1	1

Table S3. Distal eSNPs found in previous studies. SNPs with distal association to one or more genes in our work (FDR 5%) and with distal association in published studies to any gene (p<5e-08). The SNP positions are based on hg 19. "# genes" denotes the number of genes in the current study, while "# genes comp" denotes the number of genes discovered in the comparison studies.
